# A Simple Method for Differentiating Complicated Parapneumonic Effusion/Empyema from Parapneumonic Effusion Using the Split Pleura Sign and the Amount of Pleural Effusion on Thoracic CT

**DOI:** 10.1371/journal.pone.0130141

**Published:** 2015-06-15

**Authors:** Naoki Tsujimoto, Takeshi Saraya, Richard W. Light, Yayoi Tsukahara, Takashi Koide, Daisuke Kurai, Haruyuki Ishii, Hirokazu Kimura, Hajime Goto, Hajime Takizawa

**Affiliations:** 1 Department of Respiratory Medicine, Kyorin University School of Medicine, Tokyo, Japan; 2 Division of Allergy/Pulmonary/Critical Care, Vanderbilt University Medical Center, Nashville, Tennessee, United States of America; 3 Department of Radiology, Kyorin University School of Medicine, Tokyo, Japan; 4 Infectious Disease Surveillance Center, National Institute of Infectious Diseases, Tokyo, Japan; Fundación Jimenez Diaz, SPAIN

## Abstract

**Background:**

Pleural separation, the “split pleura” sign, has been reported in patients with empyema. However, the diagnostic yield of the split pleura sign for complicated parapneumonic effusion (CPPE)/empyema and its utility for differentiating CPPE/empyema from parapneumonic effusion (PPE) remains unclear. This differentiation is important because CPPE/empyema patients need thoracic drainage. In this regard, the aim of this study was to develop a simple method to distinguish CPPE/empyema from PPE using computed tomography (CT) focusing on the split pleura sign, fluid attenuation values (HU: Hounsfield units), and amount of fluid collection measured on thoracic CT prior to diagnostic thoracentesis.

**Methods:**

A total of 83 consecutive patients who underwent chest CT and were diagnosed with CPPE (n=18)/empyema (n=18) or PPE (n=47) based on the diagnostic thoracentesis were retrospectively analyzed.

**Results:**

On univariate analysis, the split pleura sign (odds ratio (OR), 12.1; *p*<0.001), total amount of pleural effusion (≥30 mm) (OR, 6.13; *p*<0.001), HU value≥10 (OR, 5.94; *p*=0.001), and the presence of septum (OR, 6.43; *p*=0.018), atelectasis (OR, 6.83; *p*=0.002), or air (OR, 9.90; *p*=0.002) in pleural fluid were significantly higher in the CPPE/empyema group than in the PPE group. On multivariate analysis, only the split pleura sign (hazard ratio (HR), 6.70; 95% confidence interval (CI), 1.91-23.5; *p*=0.003) and total amount of pleural effusion (≥30 mm) on thoracic CT (HR, 7.48; 95%CI, 1.76-31.8; *p*=0.006) were risk factors for empyema. Sensitivity, specificity, positive predictive value, and negative predictive value of the presence of both split pleura sign and total amount of pleural effusion (≥30 mm) on thoracic CT for CPPE/empyema were 79.4%, 80.9%, 75%, and 84.4%, respectively, with an area under the curve of 0.801 on receiver operating characteristic curve analysis.

**Conclusion:**

This study showed a high diagnostic yield of the split pleura sign and total amount of pleural fluid (≥30 mm) on thoracic CT that is useful and simple for discriminating between CPPE/empyema and PPE prior to diagnostic thoracentesis.

## Introduction

Before diagnostic thoracentesis, pleural infection should be suspected in all patients with pneumonia persistent fever, and elevation of serum inflammatory markers such as C-reactive protein and white blood cell count. However, those clinical findings do not always indicate complicated parapneumonic effusion (CPPE)/empyema rather than parapneumonic pleural effusion (PPE). Among patients with CPPE/empyema, the frequency of surgery ranges from 15% [1] to 68% [2] and the mortality rate in patients with empyema is 15–20% [3–5]. Rapid recognition of CPPE/empyema is thus crucial to successful treatment. In this regard, thoracic computed tomography (CT) could play a pivotal role in differentiating between CPPE/empyema and PPE. The split pleura sign has been considered a diagnostic sign for empyema. However, no reports have evaluated the use of the split pleura sign to differentiate CPPE/empyema from PPE. These two clinical entities are sequential conditions, which leads to difficulty in assessing the diagnostic yield of the split pleura sign. We therefore undertook a retrospective study to evaluate the utility of the split pleura sign and total amount of pleural effusion on thoracic CT for differentiating between CPPE/empyema and PPE.

## Materials and Methods

This retrospective study was approved by the Ethics Board of Kyorin University (number: H26-032) (Mitaka, Tokyo, Japan). All patients were referred to our respiratory department in outpatient or inpatient settings in Mitaka City, Tokyo, Japan, between May 2006 and May 2014. No informed consent was required for this study, but patient records and information were anonymized and de-identified prior to analysis. The definition of CPPE/empyema was based on Light’s criteria (including only classes 6 and 7) [6] and category 3 or 4 of the American College of Chest Physicians consensus [7]. To be enrolled in the study, patients had to be older than 15 years and show pleural effusion on thoracic CT. This single-institution study retrospectively assessed patients who satisfied at least one of the criteria for CPPE or empyema mentioned above. PPE was defined as a case with clinical and radiological improvement after initiation of antibiotic therapy regardless of the presence of pneumonia and which satisfied none of the criteria for CPPE/empyema. Furthermore, patients with other etiologies for exudative pleural effusion, such as cytologically confirmed malignant pleural effusion (mesothelioma, lung cancer, and other metastatic cancers) and collagen vascular diseases or drug-associated pleural effusion or effusions of unknown etiology were not enrolled in this study. All laboratory data including thoracentesis and thoracic CT were obtained within 48 h after admission or on the same day in outpatient settings. Three respiratory physicians and one radiologist with 10 years of experience, all of whom were blinded to the clinical findings of patients, reviewed high-resolution CT (HRCT) findings independently and reached decisions by consensus.

### Measurement of each finding on thoracic CT

**Fig 1:**
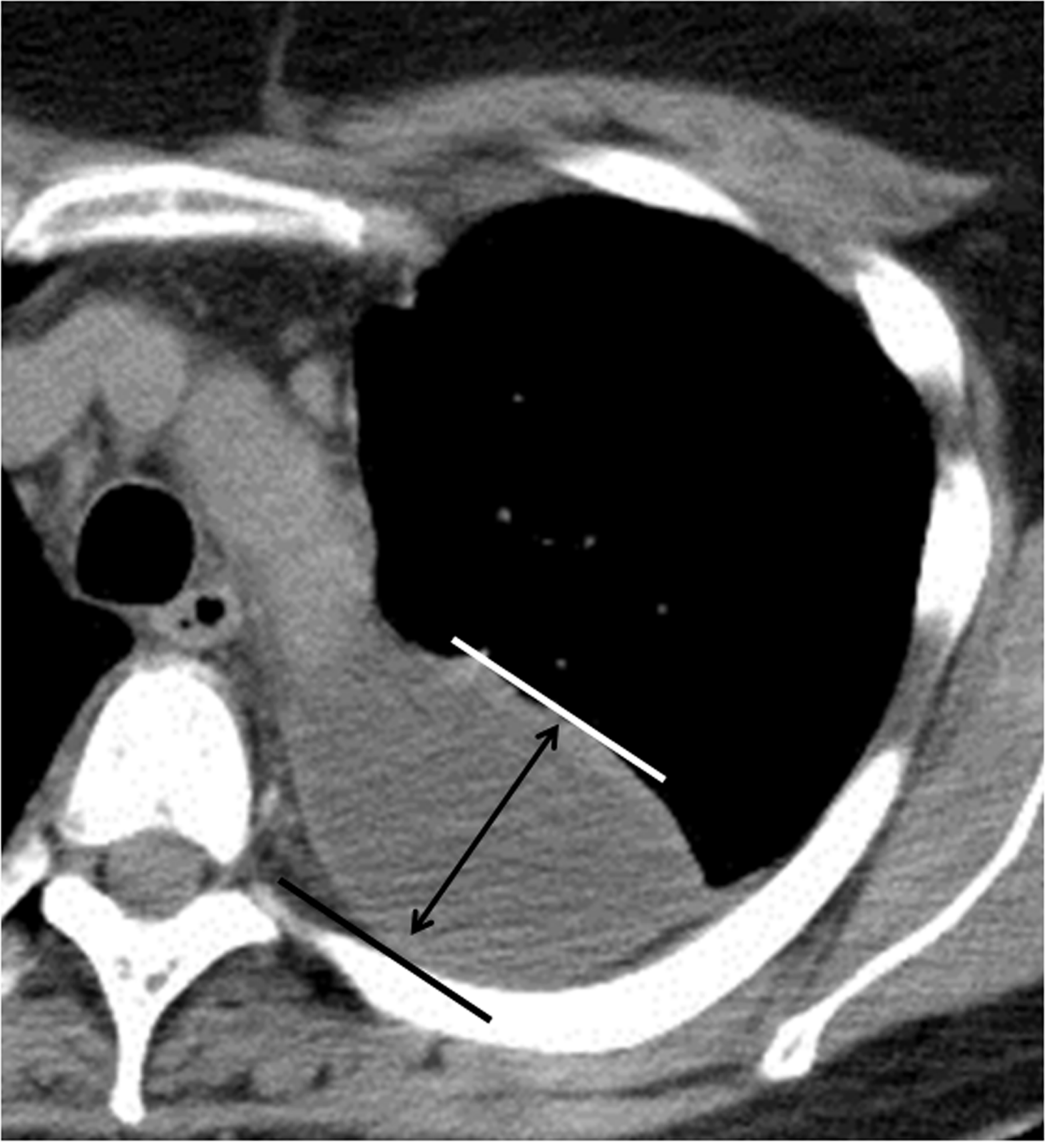
Measurement of total amount of pleural effusion. The amount of pleural effusion is calculated from the distance between the parietal and visceral pleura layers by drawing a vertical line (Fig 1).

**Fig 2:**
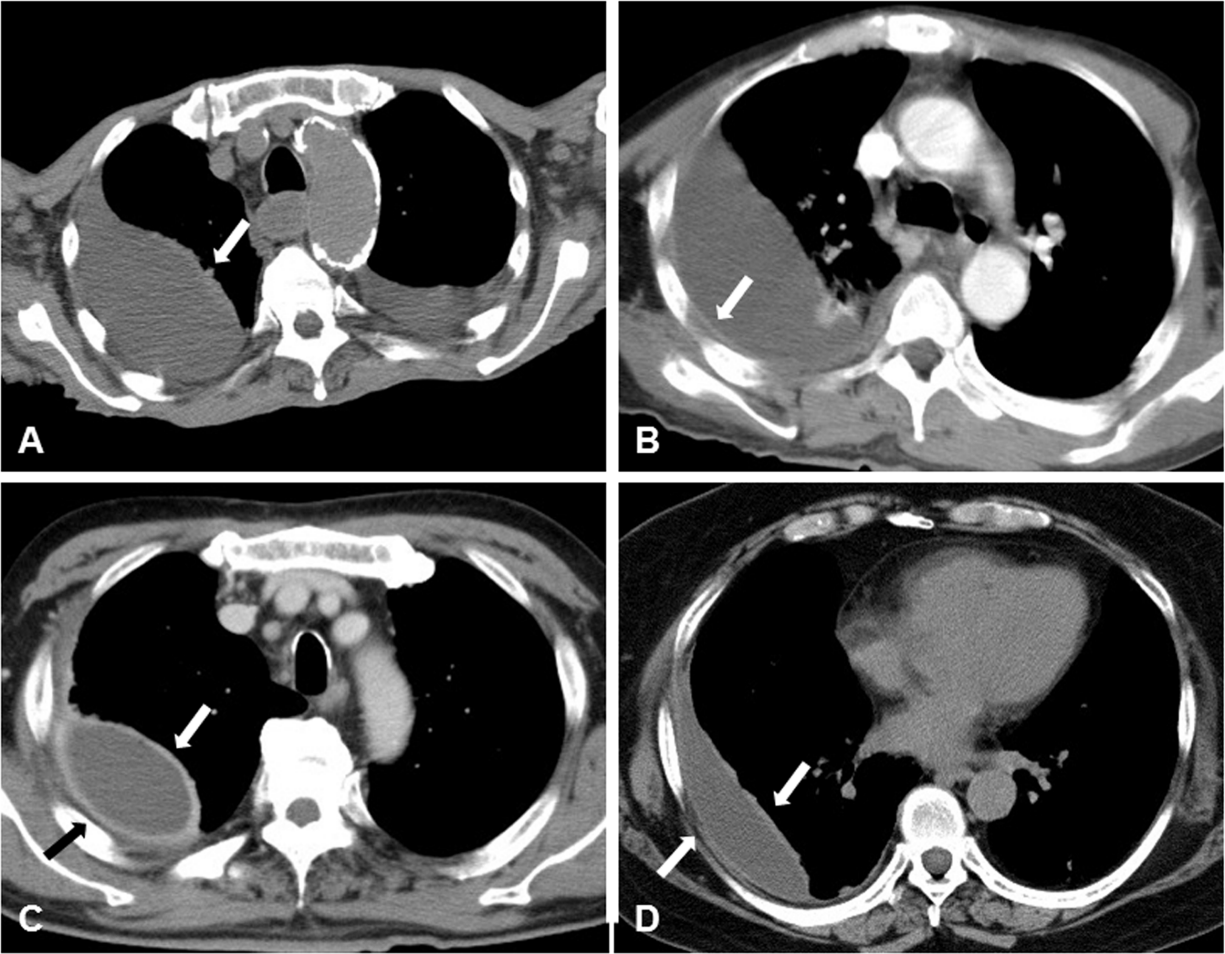
Representative figures for hemi-split pleura sign (A, B) and split pleura sign (C, D). Thickened visceral or parietal pleura on thoracic CT show the “hemi-split pleura sign” (A, B). When both pleura are thickened and separated by effusion, this is defined as the “split pleura sign” (C, D).

The amount of pleural effusion was semiquantitated as the distance between layers of parietal and visceral pleura by drawing a vertical line (Fig 1). Thickening of the visceral or parietal pleura was defined as pleura visible on thoracic CT. If thickening of either the visceral or parietal pleura was noted on thoracic CT, this was called the “hemi-split pleura sign” (Fig 2A and 2B), and if both layers of pleura were thickened and separated by effusion, this was defined as the “split pleura sign” (Fig 2C and 2D). Both pleura signs were used and evaluated even with non-enhanced CT. Hounsfield unit (HU) values of pleural fluid were assessed at three slices not adjacent to ribs, lung parenchyma, or areas of pleural thickening. Pleural effusion showing septal walls was called septated pleural effusion, and the presence of multiple septa was considered multiloculated effusion.

### Laboratory discrimination between CPPE/empyema and PPE

Correlations between serum and pleural inflammatory markers were analyzed together with correlations among pleural parameters. Predictive parameters for CPPE/empyema were extracted, and a receiver operating characteristic curve was constructed.

### Statistical analysis

Numeric data were evaluated for normal distribution and for equal variance using the Kolmogorov-Smirnov test and Levene’s median test, respectively. Categorical data are presented as percentages of the total or numerically, as appropriate. Statistical comparisons of nonparametric data were performed using the Mann-Whitney test. Comparisons of categorical data were made with Pearson’s chi-square test. Logistic regression modelling was used for uni- and multivariate analyses to identify risk factors for CPPE/empyema. Receiver-operator characteristic (ROC) curves defining the sensitivity and specificity for diagnosing empyema were constructed for both parameters (split-pleura sign and amount of pleural effusion) on chest CT. All tests were two-sided. Significance was indicated by values of p<0.05. Data were analyzed using SPSS version 19.0 software for Windows.

## Results

### Clinical characteristics of the CPPE/empyema and PPE groups

A total of 36 patients had CPPE/empyema (male, n = 30; female, n = 6) and 47 had PPE (male, n = 40; female, n = 7). Characteristics of the two groups are shown in Table 1. Mean (±standard deviation (SD)) age was 71.6±10.6 years (range, 49–94 years) for the CPPE/empyema group and 68.0±12.3 years (range, 41–88 years) for the PPE group. Groups were similar in age, male-to-female ratio, and proportion of patients who had underlying diseases or symptoms. Overall 8 patients had COPD, but only two were taking corticosteroids. In both groups, main symptoms on admission to hospital were pyrexia, chest pain, productive cough, and dyspnea, showing no significant differences between groups. The proportions of smokers and alcohol drinkers were comparable between groups, and time from initial onset to visiting our hospital did not differ significantly between groups (CPPE/empyema group, 13.0±15.3 days; PPE group, 8.6±8.4 days) (Table 1).

**Table 1:** Baseline characteristics of the CPPE/Empyema and PPE groups.

		CPPE/Empyema (n = 36)	PPE (n = 47)	p value
Age		71.6±10.6	68.0±12.3	N.S.
M:F		30:6	40:7	N.S.
Underlying disease -		4 (11.1%)	8 (17.0%)	N.S.
Underlying disease +		32 (88.9%)	39 (83.0%)	N.S.
	NIDDM type2	11 (30.6%)	14 (29.8%)	N.S
	Lung disease	10 (27.8%)	14 (29.8%)	N.S
	COPD	2	6	N.S
	Old TB	2	3	N.S
	Asthma	1	2	N.S
	Bronchiectasis	1	0	N.S
	Others	4	3	N.S
	Cardiac disease	15 (41.7%)	14 (29.8%)	N.S
	Renal disease	3 (8.3%)	6 (12.8%)	N.S
	Malignancy	9 (25.0%)	6 (12.8%)	N.S
	CVD	8 (22.2%)	4 (8.5%)	N.S
	CTD	3 (8.3%)	0 (0%)	N.S
Symptom -		3 (8.3%)	4 (8.5%)	N.S.
Symptom +		33 (91.7%)	43 (91.5%)	N.S.
	Pyrexia	21 (58.3%)	31 (70.5%)	N.S
	BT (°C)	37.5±1.1	37.5±1.0	N.S.
	Chest pain	15 (41.7%)	24 (51.0%)	N.S.
	Productive cough	14 (38.9%)	15 (31.9%)	N.S.
	Dyspnea	9 (25.0%)	8 (17.0%)	N.S.
Smoking		22 (61.1%)	27 (57.4%)	N.S.
Pack/years		29.1±31.1	34.2±28.2	N.S
Alcohol drinking		18 (50.0%)	17 (36.2%)	N.S.
The time from initial onset		13.0±15.3	8.6±8.4	N.S.

The data of the CPPE/empyema and PPE group are compared using Pearson’s χ^2^ test or the Mann-Whitney test. Data are presented as means±SD.

BT: body temperature, CPPE: complicated parapneumonic effusion, CTD: connective tissue disease, CVD: cerebral vascular disease, NIDDM: non-insulin-dependent diabetes mellitus, N.S: not significant, PPE: parapneumonic effusion

### Laboratory findings and diagnostic thoracentesis on admission

Serum lactase dehydrogenase (LDH) level was significantly lower in the CPPE/empyema group (210±125 IU/L) than in the PPE group (254±130 IU/L; *p* = 0.033) (Table 2), whereas serum white blood cell (WBC) count and C-reactive protein (CRP) level were not significantly different between groups (WBC: 13,400±6,540/μL vs. 14,800±13,600/μL; CRP: 20.4±18.1 mg/dL vs. 18.4±11.0 mg/dL).

**Table 2:** Laboratory findings of blood and pleural fluid in the CPPE/empyema and PPE groups.

Laboratory data			CPPE/empyema (n = 36)	PPE (n = 47)	*p* value
Blood					
	WBC(/μL)		13400±6540	14800±13600	N.S.
		Neut(%)	82.9±6.6	74.8±19.4	N.S
		Lym(%)	8.1±5.1	10.8±6.8	N.S
	TP(g/dL)		6.5±1.0	6.7±1.0	N.S.
	LDH(IU/L)		210±125	254±130	0.033
	Glucose(mg/dL)		170±129	136±48.8	N.S.
	CRP(mg/dL)		20.4±18.1	18.4±11.0	N.S.
Pleural effusion					
	pH		7.20±1.50	7.70±1.50	<0.001
	TCC (/μL)		156000±272000	3120±5010	<0.001
		Neut(%)	88.4±18.4	52.2±35.2	<0.001
		Lym(%)	3.8±3.1	29.8±28.4	<0.001
	TP(g/dL)		4.1±1.4	4.2±2.1	N.S.
	LDH(IU/L)		9600±13800	821±666	<0.001
	Glucose(mg/dL)		59.2±77.0	92.1±58.5	0.003
	ADA(U/L)		75.5±80.5	28.6±23.0	0.003
Pleural effusion TP/serum TP			0.6±0.2	0.6±0.3	N.S.
Pleural effusion LDH/serum LDH			53.3±79.3	4.3±3.2	<0.001

ADA: adenosine deaminase, CRP: C-reactive protein, LDH: lactase dehydrogenase, Lym: lymphocyte, Neut: neutrophil, TP: total protein, TCC: total cell count, WBC: white blood cell.

Diagnostic thoracentesis showed that the CPPE/empyema group had significantly higher total cell count (TCC) (156,000±272,000 cells vs. 3,120±5,010 cells/μL; *p*<0.001), and levels of LDH (9,600±13,800 IU/L vs. 821±666 IU/L; *p*<0.001) and adenosine deaminase (ADA) (75.5±80.5 U/L vs. 28.6±23.0 U/L; *p* = 0.003) compared to the PPE group. In addition, pleural fluid glucose levels (59.2±77.0 mg/dL vs. 92.1±58.5 mg/dL; *p* = 0.003) and pH (7.20±1.50 vs. 7.70±1.50; *p*<0.001) were significantly lower in the CPPE/empyema group (Table 2). Furthermore, the frequency of positive pleural fluid culture for bacteria was significantly higher in the CPPE/empyema group (n = 25, 69.4%) than in the PPE (0%; *p*<0.001) group. Among the 25 patients in the CPPE/empyema group, the number of single or mixed infections was 15 and 10, respectively.

**Fig 3:**
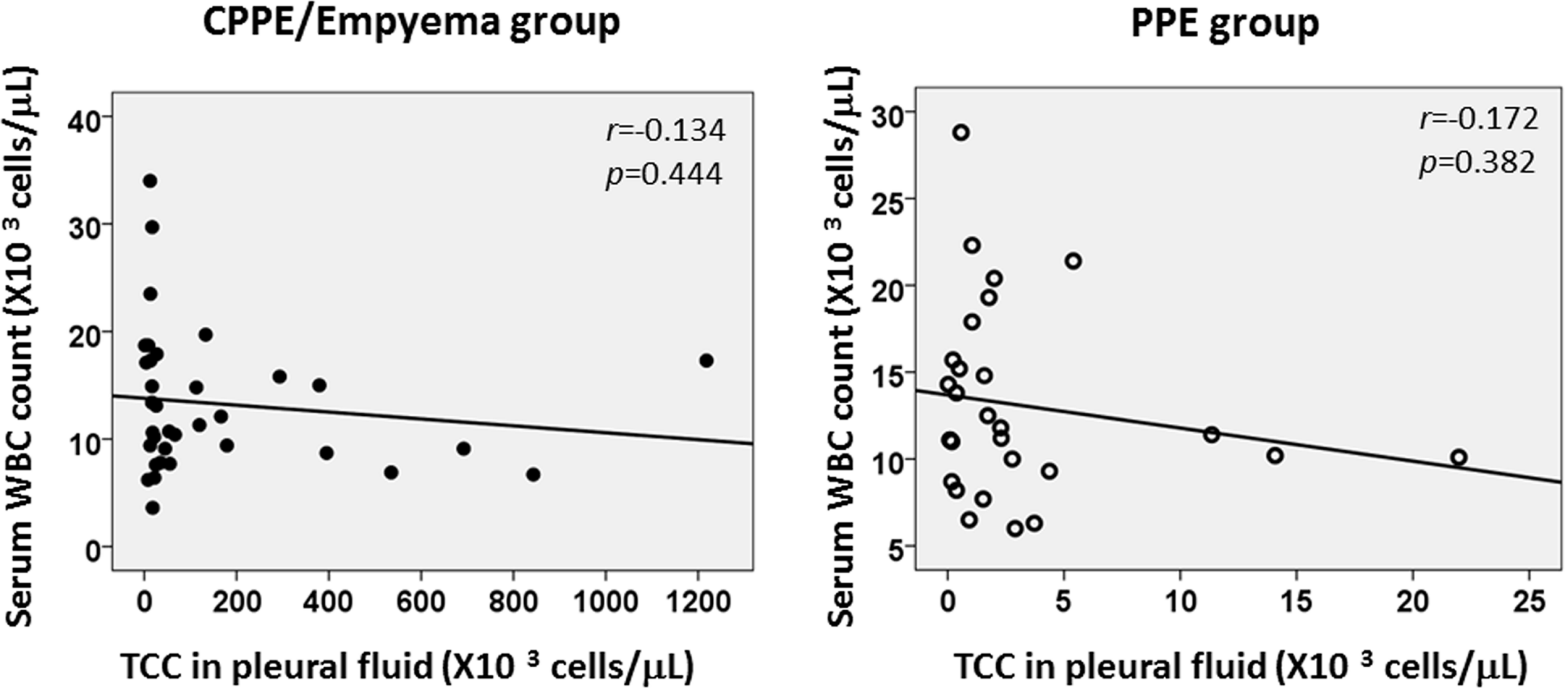
Correlation between serum WBC counts and pleural fluid TCC in the CPPE/empyema and PPE groups. No apparent associations are seen between serum WBC counts and pleural fluid TCC in the CPPE/empyema group (r = -0.134, *p* = 0.444) and PPE group (r = -0.172, *p* = 0.382). CPPE: complicated parapneumonic effusion, PPE: parapneumonic effusion, TCC: total cell count, WBC: white blood cell count.

**Fig 4:**
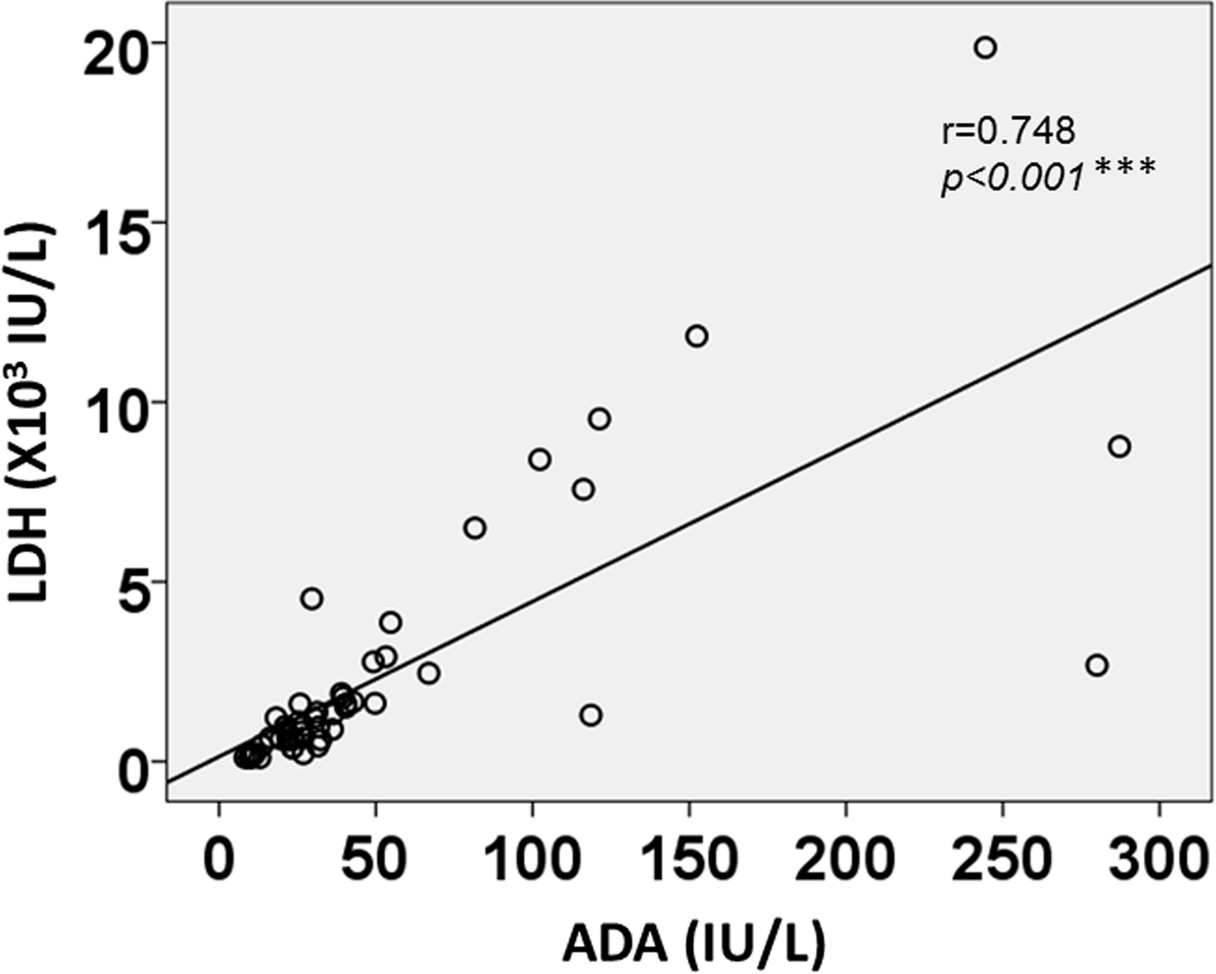
Correlation between pleural fluid LDH and ADA levels using combined data from the CPPE/empyema and PPE groups. An intense, strongly positive correlation (r = 0.748, *p*<0.001) is noted between LDH and ADA levels. ADA: adenosine deaminase, CPPE: complicated parapneumonic effusion, LDH: lactase dehydrogenase, PPE: parapneumonic effusion.

No significant associations were apparent between serum WBC count and pleural fluid TCC (Fig 3), suggesting that systemic inflammation and/or inflammatory markers do not always reflect local inflammation in the thoracic cavity. Interestingly, pleural fluid LDH and ADA levels using the combined data of both groups showed a strong positive correlation (r = 0.748, *p*<0.001) (Fig 4).

### Radiological findings

On univariate analysis, the split pleura sign was significantly more prevalent in the CPPE/empyema group (80.6%) than in the PPE group (25.5%; *p*<0.001), while frequencies of the hemi-split pleura sign, multiple lesions, and calcification were comparable (Table 3). Comparing data between the CPPE/empyema and PPE groups, HU value (15.4±8.5 vs. 9.3±8.8; *p* = 0.001), the proportion of patients with total amount of fluid ≥30 mm (72.2% vs. 29.8%; *p*<0.001), thickness of visceral pleura (4.4±3.8 mm vs. 1.1±2.0 mm; *p*<0.001), presence of a septated lesion (22.2% vs. 4.3%; *p* = 0.018), atelectasis (91.7% vs. 61.7%; *p* = 0.002), and air in pleural fluid (30.6% vs. 4.3%; *p* = 0.002) were all significantly higher in the CPPE/empyema group. Conversely, pneumonia was significantly more common in the PPE group than in the CPPE/empyema group (Table 3).

**Table 3:** Radiological findings on thoracic CT in the CPPE/empyema and PPE groups.

	CPPE/empyema(n = 36)	PPE(n = 47)	Odds ratio	*p* value
Split pleura sign	29 (80.6%)	12 (25.5%)	12.1	<0.001
Hemi-split pleura sign	5 (13.9%)	4 (8.5%)	1.73	N.S.
Hounsfield Unit (HU) ≥10	31 (86.1%)	24 (51.1%)	5.94	0.001
Total amount of PE (≥30mm)	26 (72.2%)	14 (29.8%)	6.13	<0.001
Thickness of visceral pleura≥2mm	33 (91.7%)	20 (42.6%)	14.9	<0.001
Septated lesion	8 (22.2%)	2 (4.3%)	6.43	0.018
Multiple lesion	15 (41.7%)	10 (21.3%)	2.64	N.S.
Atelectasis	33 (91.7%)	29 (61.7%)	6.83	0.002
Calcification	8 (22.2%)	6 (12.8%)	2.00	N.S.
Air in pleural effusion	11 (30.6%)	2 (4.3%)	9.90	0.002
Pneumonia	17 (47.2%)	39 (83.0%)	0.19	0.002

CPPE: complicated parapneumonic effusion, PPE: parapneumonic effusion

### Correlations among radiological parameters

**Fig 5:**
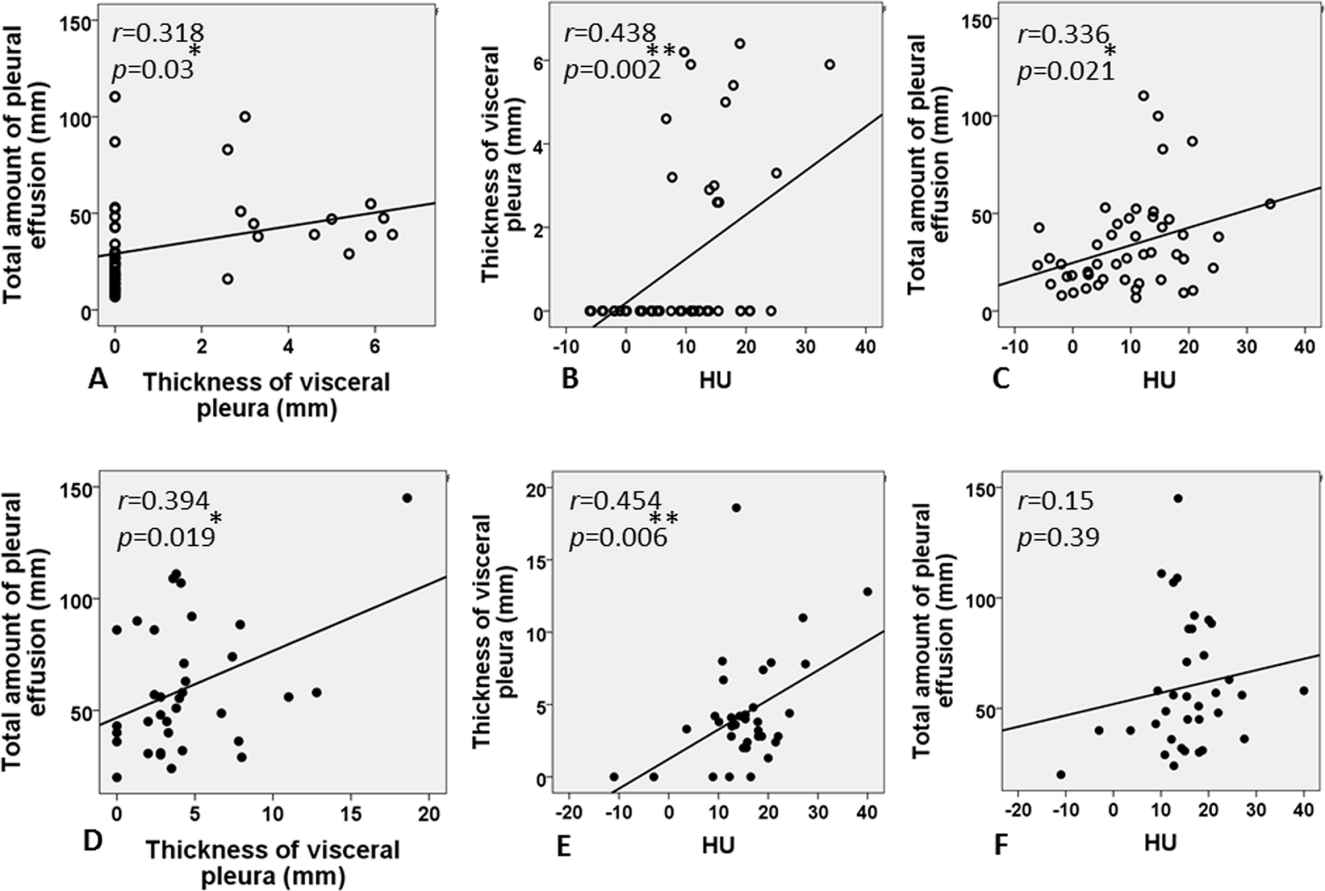
Correlations among radiological parameters in both PPE (A-C) and CPPE/empyema groups (D-F). A significant correlation between total amount of fluid and thickness of the visceral pleura is seen in both CPPE/empyema (r = 0.394, *p* = 0.019) and PPE groups (r = 0.318, *p* = 0.03). Similarly, the thickness of visceral pleura and HU (Hounsfield units) value show moderate positive correlations in both groups (CPPE/empyema group: r = 0.454, *p* = 0.006; PPE group: r = 0.438, *p* = 0.002). A significant correlation between total amount of pleural fluid and HU value is seen in the PPE group, but not in the CPPE/empyema group.

Among the parameters obtained from radiological findings, correlations were assessed using total amount of fluid (mm), thickness of visceral pleura (mm), and HU value (Fig 5). The correlation between total amount of fluid and thickness of the visceral pleura was significant in both the CPPE/empyema (r = 0.394; *p* = 0.019) and PPE groups (r = 0.318; *p* = 0.03). Similarly, the thickness of visceral pleura and HU level showed a significant moderately positive correlation in both groups (CPPE/empyema group: r = 0.454, *p* = 0.006; PPE group: r = 0.438, *p* = 0.002). A significant correlation was seen between total amount of pleural fluid and HU value in the PPE group, but not in the CPPE/empyema group.

### Predictive factors for CPPE/empyema

Logistic regression analysis was performed using the parameters extracted on univariate analysis. Only the split pleura sign (hazard ratio (HR), 6.70, 95% confidence interval (CI), 1.91–23.5; *p* = 0.003) and amount of fluid larger than 30 mm (HR, 7.48; 95%CI, 1.76–31.8; *p* = 0.006), but not HU (HR, 1.00; 95%CI, 0.93–1.08; *p* = 0.976), were significant predictive factors for CPPE/empyema (Table 4).

**Table 4:** Multivariate analysis for factors predictive of CPPE/empyema.

	HR (95% CI)	*p* value
Split pleura sign	6.70 (1.91–23.5)	0.003
Total amount of pleural effusion (≥30mm)	7.48 (1.76–31.8)	0.006
Hounsfield Unit (HU)	1.00 (0.93–1.08)	0.976

CPPE: complicated parapneumonic effusion, HR: hazard ratio

### Receiver operating characteristic curve using two factors

**Fig 6:**
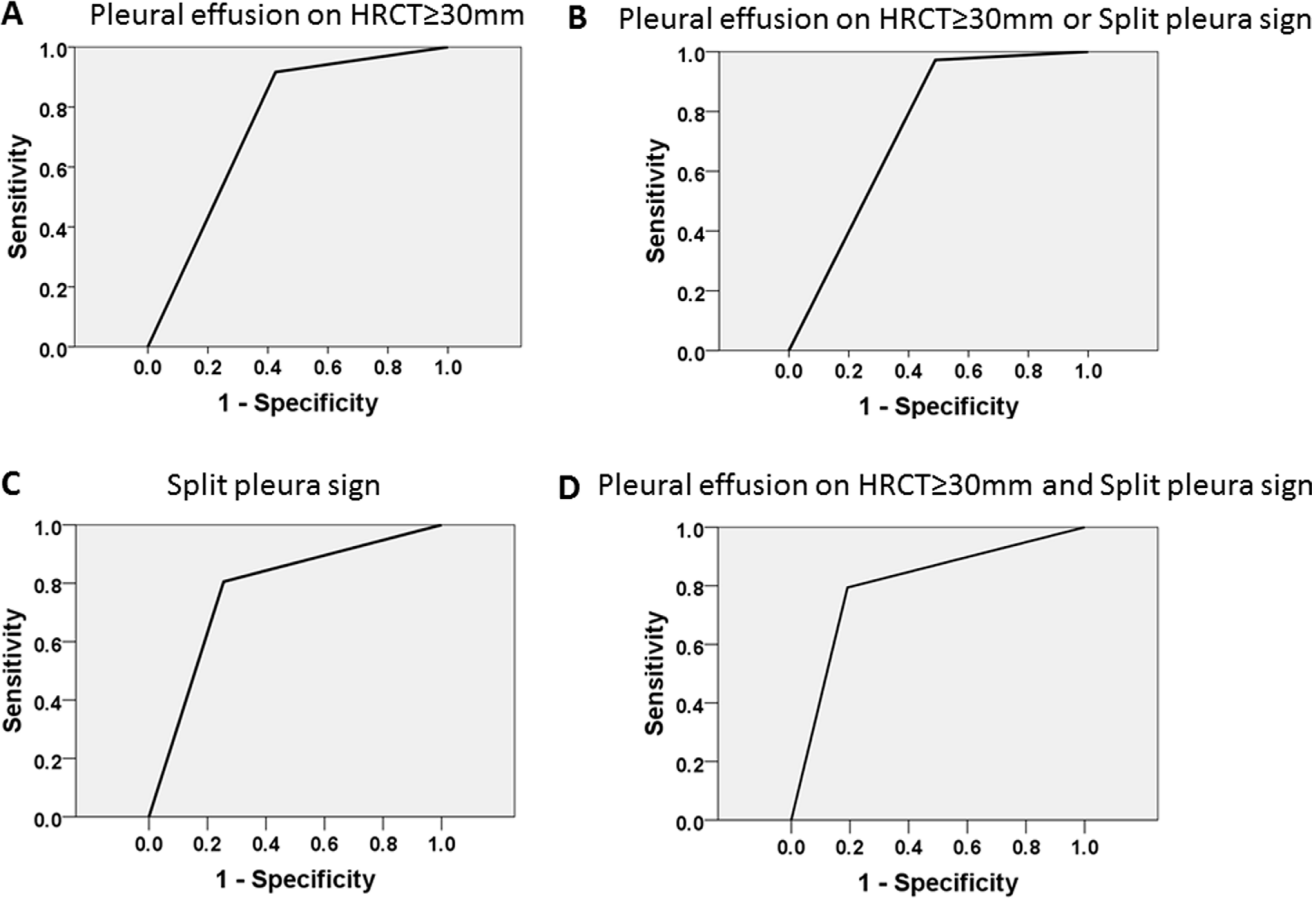
Diagnostic accuracy for CPPE/empyema using two parameters. Receiver-operator characteristic (ROC) curve using the split pleura sign and total amount of pleural effusion (≥30 mm). The split pleura sign (C) shows 80.6% sensitivity, 74.5% specificity, a positive predictive value of 74.5%, and a negative predictive value of 70.7%, with an area under the curve of 0.775. The presence of both split pleura sign and total amount of pleural effusion (≥30 mm) (D) shows 79.4% sensitivity, 80.9% specificity, a positive predictive value of 75%, and a negative predictive value of 84.4%, with an area under the curve of 0.801. A: pleural effusion ≥30 mL; B: pleural effusion ≥30 mL or split pleura sign; C: split pleural sign; D: total amount of pleural effusion ≥30 mL and split pleura sign.

ROC curve analysis was performed for the two parameters (split pleura sign and amount of pleural effusion ≥30 mm) identified by multivariate analysis. The split pleura sign was better than amount of pleural effusion ≥30 mm for diagnosing CPPE/empyema, with 80.6% sensitivity, 74.5% specificity, a positive predictive value of 74.5%, a negative predictive value of 70.7%, and an area under the curve of 0.775, and the presence of both split pleura sign and total amount of pleural effusion (≥30 mm) (D) shows 79.4% sensitivity, 80.9% specificity, a positive predictive value of 75%, and a negative predictive value of 84.4%, with an area under the curve of 0.801 (Table 5; Fig 6).

**Table 5:** Diagnostic accuracies of the two risk factors for CPPE/empyema.

	Sensitivity	Specificity	AUC	
PE ≥30 mm	91.2	57.4	0.743	*p*<0.001(95%CI: 0.635–0.851)
Split pleura sign	80.6	74.5	0.775	*p*<0.001(95%CI: 0.671–0.880)
PE ≥30 mm or Split pleura sign	97.1	51.1	0.741	*p*<0.001(95%CI: 0.634–0.848)
PE ≥30 mm and Split pleura sign	79.4	80.9	0.801	*p*<0.001(95%CI: 0.699–0.904)

AUC: area uncer the curve, CPPE: complicated parapneumonic effusion, PE: pleural effusion

## Discussion

Discriminating CPPE/empyema from PPE is often quite difficult, especially before diagnostic thoracentesis, because of the clinical similarities of these two sequential conditions, as seen in the present study (Tables 1 and 2). Interestingly, the time from initial onset to the first visit to hospital and the proportions of underlying diseases did not differ significantly between the CPPE/empyema and PPE groups. Furthermore, no significant correlation between systemic inflammation (serum WBC or CRP levels) and local thoracic inflammation (pleural fluid TCC) was found in either group (Fig 3). Although serum LDH level was significantly higher in the PPE group than in the CPPE/empyema group, LDH levels in the pleural fluid were higher in CPPE/empyema group than that of PPE group. This indicates that pleural inflammation is not necessarily reflected in the serum. However, some studies that examined correlations between systemic inflammation (inflammatory markers) and local inflammation (pleural cavity) using interleukin-18 [8] or soluble Fas ligand [9] found no clear evidence of positive correlations with those markers. This presents a diagnostic dilemma for physicians.

In this regard, radiological assessment for differentiating CPPE/empyema from PPE seemed to be pivotal in the diagnostic process. To the best of our knowledge, only six reports have been published regarding the split pleura sign [10–15]. Stark et al. reported that the split pleura sign was seen in 68% of pleural empyema cases [13] and is considered the most reliable CT sign helping to distinguish empyema from pulmonary abscess [13] or noninfectious pleural effusion [14]. However, no report has described the utility of the split pleura sign [15] in differentiating CPPE/empyema from PPE. From this perspective, the present series showed that the split pleura sign could be a useful marker for differentiating CPPE/empyema (HR, 6.70; 95%CI, 1.91–23.5; *p* = 0.003) from PPE. Furthermore, the amount of pleural fluid (≥30 mm) on thoracic CT was also found to be a suggestive factor for CPPE/empyema (HR, 7.48; 95%CI, 1.76–31.8; *p* = 0.006). Being aware of these two predictive factors for CPPE/empyema is a simple way for physicians to assess the probability of CPPE/empyema prior to performing diagnostic thoracentesis.

The present study has some limitations in that: 1) only 15 of 36 patients (41.7%) in the CPPE/empyema group and 8 of 47 patients (17.0%) in the PPE group underwent enhanced thoracic CT, which might have affected the results for the incidence of the split pleura sign; 2) thoracic CT might not be available in local hospitals or clinics; and 3) other pleural diseases such as malignant effusion, mesothelioma, and tuberculous pleuritis were outside of the scope of this study, and also need to be ruled out in general practice; 4) the study was retrospective. However, this study showed a strong correlation between pleural LDH and ADA levels (Fig 4) using the combined data from both CPPE/empyema and PPE groups, implying that these measurement might be useful for differentiation from the other pleural diseases described above. In conclusion, this is the first study to demonstrate the high diagnostic yield of both split pleura sign and large pleural effusion (≥30 mm) on thoracic CT for discriminating between CPPE/empyema and PPE. Early recognition of CPPE/empyema using those signs could help decrease morbidity and mortality.
